# Assessment of the Effect of Kinesiology Taping on Scar Treatment in Children

**DOI:** 10.3390/clinpract15070131

**Published:** 2025-07-14

**Authors:** Justyna Pogorzelska, Agata Michalska, Anna Zmyślna

**Affiliations:** 1 Institute of Physiotherapy, Faculty of Medicine and Health Sciences, Jan Kochanowski University, 25-317 Kielce, Poland; michalska.agata@ujk.edu.pl (A.M.); anna.zmyslna@ujk.edu.pl (A.Z.); 2 Świętokrzyskie Center for Pediatrics, Regional Hospital in Kielce, 25-736 Kielce, Poland

**Keywords:** keloid, hypertrophic scar, postoperative adhesion, kinesiology taping, ultrasound, the Vancouver Scar Scale

## Abstract

**Background**: The consequences of injuries resulting from accidents are among the most common health disorders in children. A scar forms at the site of the injury. In the treatment of scars, not all methods used in adults can be used in children. The authors attempted to assess the effectiveness of using KT kinesiology taping on scars in children. The aim of the work is to assess the effect of KT on the treatment of keloid, hypertrophic scars, and postoperative adhesions in children. **Methods**: The study included 30 patients aged 4 to 10 years. The subjects were divided into three groups: group G1-9 patients with keloid scars, group G2-14 with hypertrophic scars, group G3-7 with postoperative adhesions. The patients underwent kinesiology taping for 8 weeks. The analyzed parameters were determined using the VSS scale and ultrasonography. **Results**: The analysis of the VSS scale results in relation to the type of scars showed a significant (*p* < 0.001) downward trend in the measured parameters for keloid and hypertrophic scars. Analysis of ultrasound results in relation to the type of scars showed a significant (*p* < 0.001) downward trend in the measured parameters, comparing parameters I and II for all types of scars. **Conclusions**: Kinesiology taping significantly changes the following scar parameters: deformability, pigmentation, and perfusion in the case of keloid and hypertrophic scars.

## 1. Introduction

Every organism in which skin and the underlying tissues have been damaged as a result of a disease, mechanical trauma or surgical intervention strives to restore their continuity as soon as possible [1]. At the site of the injury, a replacement tissue, namely a scar, is formed. Despite the long-term interest in the issue of scars, knowledge about the process of their formation and treatment continues to be the research topic. It remains in the areas of interest of many fields of medicine, including dermatology, surgery, and physiotherapy. The problem concerns both the issue of pathogenesis, clinical evaluation, and the selection of the optimum treatment method [2].

In the literature, there are many reliable and recommended methods of dealing with scars [3]. However, there is no ‘gold standard’ for scar treatment that could at the same time affect individual clinical parameters of the scar considering its thickness/height, color, pliability/tenacity, pigmentation, and warming [4].

In the treatment of the scars with a tendency to overgrow, both pharmacological and physical agents and surgical procedures are used [5,6]. Systematizing, invasive, and non-invasive procedures can be distinguished. Invasive treatment is most often surgical excision and re-suturing of the wound. Basically, this should only be considered when re-intervention can provide better healing than the first time (less inflammation, better technique). The surgical method, despite its effectiveness and frequency of use, is burdened with a high risk of complications (relapse of 45 to 100% of cases) [7]. Another form is the treatment with corticosteroids applied in the form of injections, by injecting the preparation precisely into the treated lesion or under the scar. Susceptibility of the scars to such treatment is 50–100%, with 10–50% frequency of relapses; however, side effects are as follows: atrophy and thinning of the skin, and discoloration [8]. Other treatments that are recommended with variable results include botulinum toxin, interferon and bleomycin injections, as well as radiation therapy, laser therapy and cryosurgery. Non-invasive treatment usually includes pressotherapy as a preventive and therapeutic method recommended for freshly healed wounds with a tendency to scar hypertrophy, as well as a variety of creams and ointments applied topically for scars, silicone gels and dressings, antihistamines, hydrotherapy, and scar mobilization [9].

The methods that are used for the work with a scar should be determined depending on the type, age, size of the scar and the patient’s age [10]. There are plenty of scientific papers on scar management. Most of them stem from cases pertaining to adults, without taking into account pediatric patients. Scars in children grow proportionally as the child does, becoming more visible and unaesthetic as they age. Scars also pose a problem when it comes to the body’s normal growth [11]. Changes in spatial configuration of forming collagen fibers in damaged tissues may thus lead to posture problems caused by tension, discomfort, and pain during the child’s development. Therefore, the process of searching for new solutions for working with scars seems even more justified. Based on their own experience and observations, the authors have attempted to assess the effectiveness of application of KT kinesiology taping for the scars in children [11,12]. In our studies, kinesiology taping was used for hypertrophic, keloid scars and postoperative adhesions in pediatric patients. The KT method consists of using the physical properties of the tape and the appropriate method of its application. The tape used for the application has properties similar to human skin. It extends to approx. 30–40% of the resting length. The thickness of the tape is similar to that of the epidermis, which limits feeling the ‘weight’ of the tape. The tape is waterproof and air-permeable, which allows air exchange and moisture evaporation. The basic functions of the KT application include normalization of muscle tone, activation of damaged muscles, correction of joint position, elimination of stasis and lymphoedema, reduction in pain and sensory hypersensitivity in the skin and muscle, as well as correction of fascia and skin. The advantage of the KT method is low invasiveness and ease of use. Two research tools were selected to assess the effectiveness of therapy. The Vancouver Scar Scale is one of the most commonly used scales in clinical trials. It assesses four traits: perfusion, pigmentation, deformation, and height/thickness [13]. The other tool is ultrasonography, which is one of the few non-invasive methods that assesses changes in epidermis and dermis [14].

## 2. Materials and Methods

### 2.1. Material and Methods

A group of 75 patients was examined. All patients were diagnosed with scars in the form of hypertrophic scars, keloid scars, and inverted scars-adhesions. Following the eligibility stage completed by a physician specializing in general surgery, 45 patients were included in the study group. Out of this group, 15 patients were excluded during the first stage of the treatment due to patient illness, treatment discontinuation, failure to perform a follow-up examination, or a fortuitous event. A total of 30 patients were deemed eligible for other scar treatment forms unrelated to the treatment using the kinesiology tapes.

The study group consisted of 12 females and 18 males aged 4 to 10 receiving treatment and therapy services at the Swietokrzyskie Paediatrics Centre. The subjects were put into 3 groups:

- Group G1 included 9 patients diagnosed with keloid scar;
- Group G2 included 14 patients diagnosed with hypertrophic scar;
- Group G3 included 7 patients diagnosed with postoperative adhesions.

All patients were included in the study not earlier than one month and not later than one year since the wound had healed. It was linked to the scar remodeling phase, which starts dominating in the 3rd week following wound closure and may last intensely for up to 2 years. To systematize causes of injury that triggered wound formation, they were divided into incised surgical wounds that included: surgeries, removal of skin lesion, surgeries following appendicitis, bowel obstruction as reoperation, and following gastroschisis. The second group included non-surgical wounds: burns, lacerated wounds, and injuries. Size criterion was applied to ensure that the KT application area was similar. The size was limited to 15 cm length- and width-wise. Scars resulting from surgical treatments were the smallest, with their width ranging from 2 cm in width and up to 15 cm in length. The most common location of scars in the study groups was the abdomen and upper limb.

#### 2.1.1. Inclusion Criteria

- The patient’s age 4–10 years old;
- Wound healing and scar formation with no signs of cracks. The scar could only be included in the study three weeks after it had healed;
- The scar age does not exceed 1 year;
- The scar size has a length and width up to 15 cm.

#### 2.1.2. Exclusion Criteria

- Wound not healed;
- Discontinuation of treatment, failure to perform a check-up within a specified time (8 weeks from the first examination);
- A child’s intolerance of specific treatment, e.g., patch allergy/intertrigo;
- Cancer.

Kinesiology taping was applied for the scars in patients undergoing 8 weeks of treatment. The whole clinical observation was divided into three stages:

#### 2.1.3. Stage I: I Taking Measurements

- Scar assessment using: the Vancouver Scar Scale (VSS), including the following features: perfusion, height/thickness, deformability, pigmentation;
- Ultrasound assessment of the scar morphology. TOSHIBA APILO 500 ultrasound scanner (Toshiba, Tokyo, Japan) was used for measurements; in order to standardize the results of the USG, only two dimensions were specified: the thickness and width of the scar.

**Figure 1:**
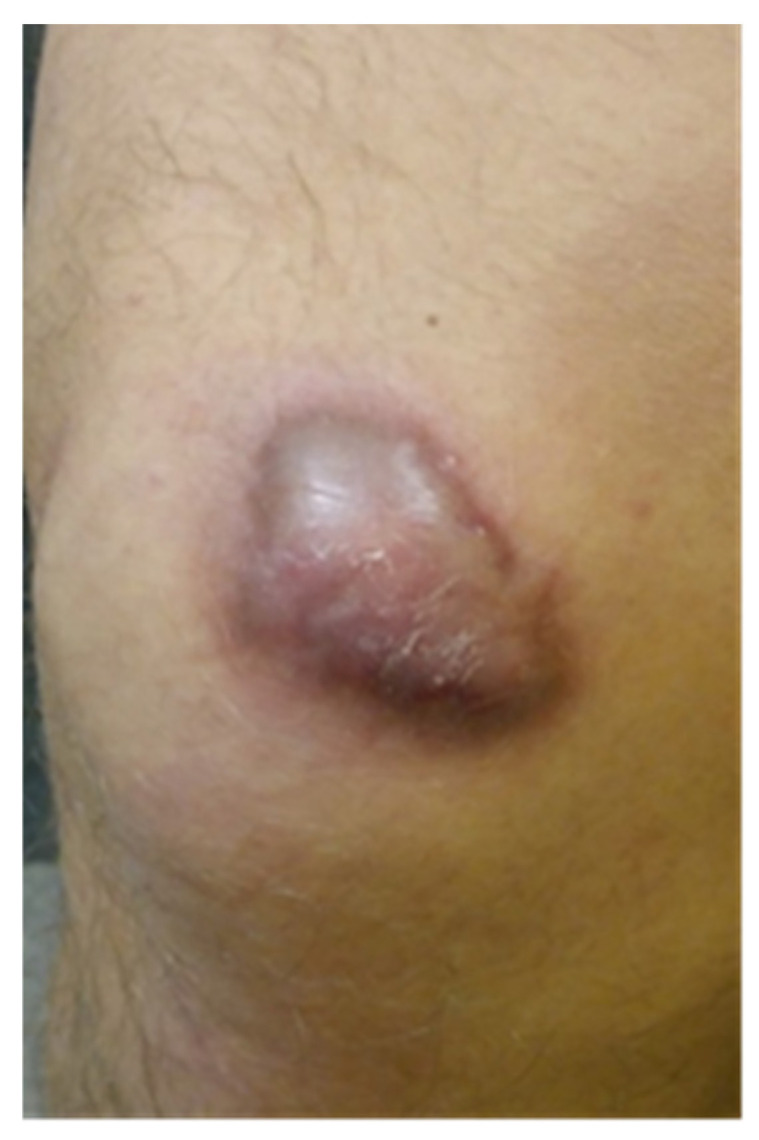
The scar before KT application (our patient)**.**

**Figure 2:**
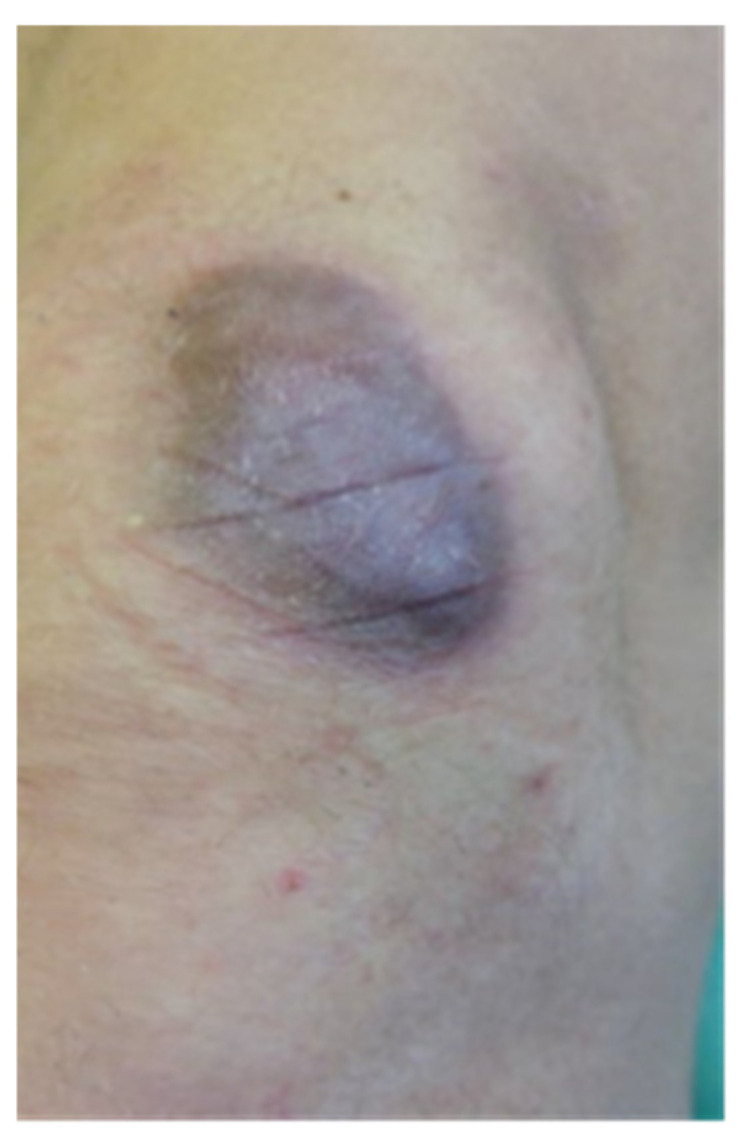
The scar after 5 days of KT application (our patient)**.**

**Figure 3:**
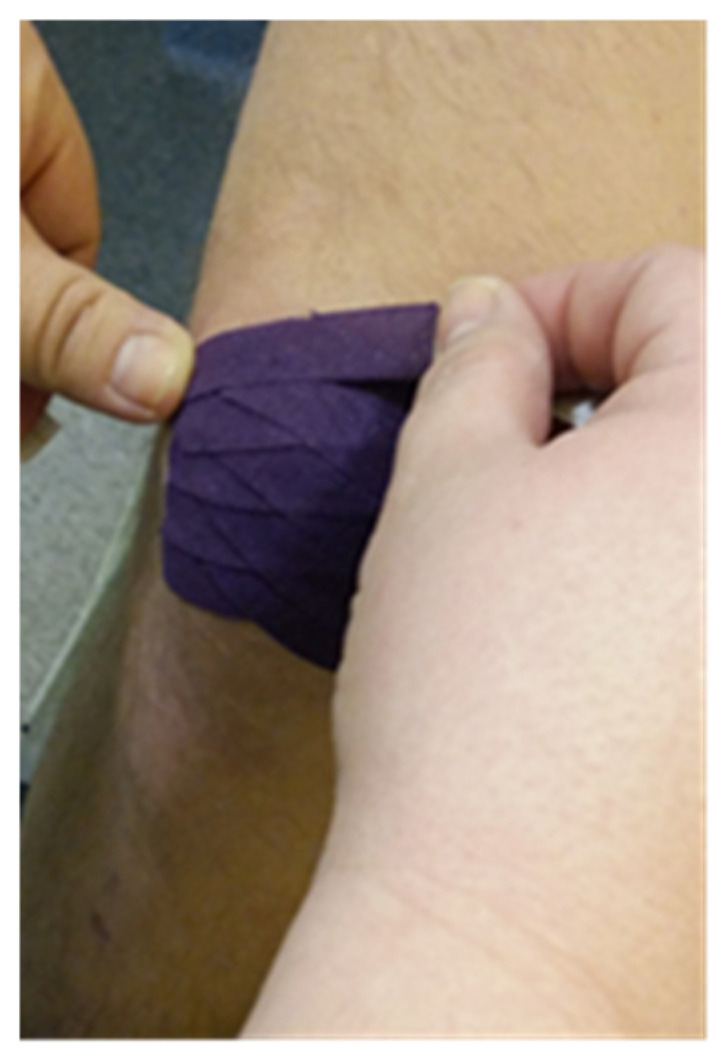
An example of the type of application (our patient)**.**

Stage II: after taking measurements with VSS and USG, KT tape for the scars was applied. The average application time of the tape to the patients’ skin was 5 days. The patients had fascial techniques applied with tape stretching from 25% to 50%. Depending on the present restrictions, the right direction of applying the kinesiology tape was selected. The anchor of the tape was placed right before the scar and the tape was stretched over the scar itself. Narrow 0.5 cm wide tape pieces, applied at a 45-degree angle towards each other (Figure 1, Figure 2 and Figure 3) were placed on the patient’s body. The therapeutic effect was the most remarkable at the point where the kinesiology tapes crossed. Two-day breaks were taken between subsequent applications to avoid unwanted allergic reactions or chafing. In total, the study lasted for 8 weeks.

Stage III: II Taking Measurements After 8 Weeks of the Treatment

- Measurements were taken using the following:
- The Vancouver Scar Scale (VSS);
- Ultrasound examination.

Imaging examination using USG and the assessment by means of the VSS before and after the KT application were performed in the same room, at a similar time of day, and by the same person. During the clinical observation, the patients did not undergo any other medical interventions.

The parents/guardians had given their written consent to conduct the tests and the treatment after being informed about the course and goals of the tests. The tests were approved by the Bioethics Committee.

## 3. Results

### 3.1. The Analysis of the Vancouver Scar Scale Scores Against the Scar Type

**Figure 4:**
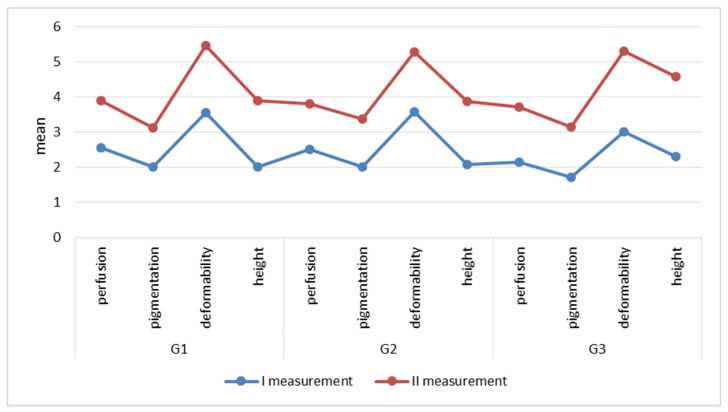
The Vancouver Scar Scale scores depending on the scar type.

Clinical evaluation of the effects of the treatment by means of the VSS showed an improvement in the quality of the scars, with the biggest positive change in relation to the deformation trait in group G1 (keloid scars) and G2 (hypertrophic scars). The mean value of the deformation trait in group G1 before the treatment was 3.56 and after 8 weeks of KT application 1.89. In group G2, before the treatment the mean value was 3.57 and after 8 weeks of KT application it was 1.7. The least positive change was recorded for the height trait. In group G1, the mean value of this trait was 2.07 and after KT application it was 1.79. In group G3 (postoperative adhesions), the value of this trait had not changed (before-2.29, after-2.29) (Figure 4).

The analysis of the Vancouver Scar Scale scores depending on the scar type showed a significant (*p* < 0.001) downward tendency of the measured parameters comparing the I and II measurements in the groups G1 and G2 (Table 1). In group G3, the analysis of the scores before and after treatment showed no significance (*p* > 0.05) (Table 1).

### 3.2. The Analysis of Scar Quantitative Parameters in USG Assessment Against the Scar Type

Ultrasonography was used for quantitative measurements. Two dimensions were specified: scar thickness and width. In the case of group G3, the scar thickness was measured from the skin surface deep into the tissues. The analysis of the USG results in relation to the scar type showed a significant (*p* < 0.001) downward tendency of the measured parameters, comparing I and II measurements in groups G1, G2, and G3 (Table 2).

**Figure 5:**
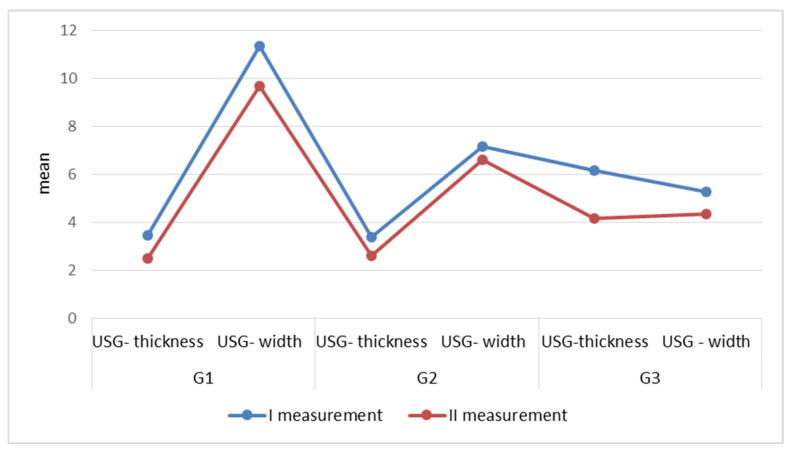
The scores of the selected scar parameters measured by USG depending on the scar type.

In group G1, the biggest change was recorded for the scar width parameter, where the mean value of this trait before the KT application was 11.36, and after 8 weeks of application, the average value decreased to 9.67. Group G2 is characterized by a similar change in the mean values of the measured parameters. In group G3, the biggest change was recorded for the parameter-scar thickness. The mean value of this trait before application was 6.17 and after 8 weeks it decreased to 4.17. The results in group G3 indicate the reasonability of application of kinesiology taping also in the case of postoperative adhesions (Figure 5).

## 4. Discussion

As has been mentioned, the scars resulting from the injury differ in their structure from the normal skin, and their morphological appearance is conditioned by the course of changes occurring within the area affected by the injury. Tissue healing can cause various types of scars, from a thin linear scar to atrophic scars, hypertrophic scars, keloids, scar contractures, or adhesions [15]. From a medical point of view, most scars do not require specialized treatment and heal without any intervention. As a result, their spontaneous reduction occurs. However, there are clinical situations in which it is necessary to initiate active therapeutic treatment [16]. This is applicable to the patients in whom the injury has led to the formation of a scar impairing everyday performance in both functional and emotional terms [17,18]. In children, postural disorders caused by tensions, discomfort, and pain in the course of the child’s development may be a threat resulting from the scars [10,19]. A large number of publications are available on the subject of using post-surgical kinesiology taping, but its application usually focuses on the problems of developing hematomas and postoperative edema [20,21,22].

As a non-invasive technique, kinesio taping can be used at any age and in many diseases. The mechanism of action of KT consists of leveraging the body’s ability to heal itself [23]. At the same time, it is assumed that applying the tape does not restrict body movements, which allows for the adjustment of disordered joint positions, pain alleviation, improved fluid flow in tissues and normalization of disordered muscle tension [23]. Moreover, applying the tape raises the skin, thus increasing the spaces in subcutaneous tissues where exteroceptors, lymphatic, and blood vessels are located. As a result, the irritation of mechanoceptors and nociceptors distributed in the skin is reduced, resulting in a decreased sensation of pain [24]. Kenzo Kase, the inventor of the method, believes that increasing the space between the skin and the fascia surrounding the muscles leads to a decrease in blood pressure and facilitates lymph flow resulting in reduced inflammation [25]. Kinesio tapes are placed on scars using the fascial technique. While soft tissue mobility at the scar site and underneath it is at risk, it may weaken motor functions and cause mobility limitations of the surrounding myofascial junctions. The flexibility and stretchiness of tapes whose properties are similar to those of human skin allow for gentle movement of the scar tissue during body moves. Gradual stretching and moving of the scar leads to its increased flexibility and improved mobility. Kinesiology tapes also stimulate cutaneous mechanoreceptors and proprioceptors which impacts the improvement of deep sensation and neuromuscular control in the scar area. It fosters normal remodeling of the scar tissue and prevents the formation of pathological scarring. There are few studies in the literature regarding the effect of kinesiology taping on scars, the majority of which apply to adult patients.

When reviewing the literature, the authors’ own studies are available [26], in which the effectiveness of kinesiology taping on a group of 54 pediatric patients was assessed. The observation of the group lasted 12 weeks, and the effects of the therapy were assessed by means of the self-designed survey questionnaire and a digital caliper. Another work is a case study of an 8-year-old female patient after a burn, in whom kinesiology taping was used in the treatment process [27]. Both studies displayed the effectiveness of KT, with the assessment based on the patient’s subjective feelings and visual assessment. Further studies concern adult patients. Goodridge S. presented a case study showing the effectiveness of kinesiology taping [28]. The author applied the kinesiology tape to a hypertrophic scar, the consequence of three surgeries, on the patient’s abdominal wall. The treatment resulted in a reduction in the scar height to 2 mm, a change in the color of the scar vascularization, increased pliability, improved motor function, and significant pain reduction. Branstiter G. presented the results of the kinesiology taping method [29]. As a result of the therapy, muscle and fascial relaxation, increase in lymph flow, and complete disappearance of edema followed, and the scar took the form of a thin, bright line. The above studies indicate that kinesiology taping can affect various scar parameters causing its reduction, discoloration, improvement of pliability, and pain reduction.

In our own studies, the possibility of using KT for keloid, hypertrophic scars and postoperative adhesions in children was presented. Applications on the scars had been used for 8 weeks and the obtained results were verified using the Vancouver Scar Scale and ultrasonography. The analysis of the Vancouver Scar Scale scores in relation to the type of scars showed a significant (*p* < 0.001) downward tendency of the measured parameters between I and II measurement for keloid and hypertrophic scars. The biggest positive change in relation to the measured parameters was obtained in terms of deformability. In the case of postoperative adhesions, the analysis of the scores before and after the treatment did not show any significance (*p* > 0.05). Ultrasonography was used for quantitative measurements. Two dimensions were specified: scar thickness and width. The analysis of USG results in relation to the scar type showed a significant (*p* < 0.001) downward tendency of the measured parameters when comparing I and II parameters for all types of scars including groups G1, G2, and G3. The existing differences in the measured parameters in the G3 group in the subjective assessment using the Vancouver scale and the objective assessment using ultrasound result from the nature of the scars. Adhesions often have the character of retracting scars, which are formed as a result of pathological connections between individual skin layers. Hence, under ultrasound control, the length and width of the scar are measured deep into the tissue. Using the Vancouver scale, the assessment of adhesions is performed only on the skin surface. The demonstrated effectiveness of kinesiology taping on postoperative adhesions in group G3 enables early interventions related to the formation of adhesions. The first postoperative adhesions are formed as early as 3 h after the surgery [30]. Therefore, the earlier preventive treatment is undertaken, the lower the possibility of secondary complications such as pain and body mechanics disorder. In comparison, KT can be included 3 weeks after the healing of the postoperative wound, while manual actions on the same scar can be undertaken only 6–8 weeks after the surgery [31].

Similar studies, which included ultrasonography and the Vancouver Scar Scale, are presented by Ahmed M. [32]. Thirty adult patients aged 20 to 45 with forearm hypertrophy scars participated in the study. The subjects were divided into two groups where for 8 weeks kinesiology taping was used in one group and deep tissue massage in the other. The obtained results show that both groups had a significant reduction in scar thickness and improved scar appearance (*p* < 0.005), and the comparison between the two groups showed that kinesiology taping is more effective than deep tissue massage.

In the scar management standards, the kinesiology taping method is not mentioned. However, with reference to the obtained results and the demonstrated effectiveness of KT, its role is worth considering in the interdisciplinary approach to scar management.

## 5. Conclusions

1. Kinesiology taping favorably changes the following scar parameters: deformation, pigmentation, and perfusion in the case of keloid and hypertrophic scars.

2. The use of kinesiology taping for keloid, hypertrophic, and postoperative scars reduces the thickness and width of the scar.

## Figures and Tables

**Table 1:** The Vancouver Scar Scale scores depending on the scar type.

Scar Type	VSS	Mean	Me	SD	Min	Max	Q1	Q3	Wilcoxon Test
G1	(I measurement)	10.11	1.05	9	9	11	11	11	Z = 2.6413 *p* = 0.0083
	(II measurement)	6.22	1.09	4	6	6	7	8	
G2	(I measurement)	10.14	1.29	7	9	11	11	11	Z = 3.2921 *p* = 0.0010
	(II measurement)	6.14	1.10	4	6	6	6.8	8	
G3	(I measurement)	9.14	1.95	6	8	10	11	11	Z = 0.9487 *p* = 0.3428
	(II measurement)	7.57	1.90	5	6.5	8	8	11	

**Table 2:** The analysis of measurements by means of USG depending on the scar type.

Scar Type	USG I and II Measurement	Mean	Me	SD	Min	Max	Q1	Q3	Wilcoxon Test
G1	thickness (I measurement)	3.46	3.50	0.46	2.70	4.00	3.00	3.80	Z = 2.6132 *p* = 0.0090
	thickness (II measurement)	2.50	2.00	0.76	1.60	3.50	2.00	3.20	
	width (I measurement)	11.36	12.00	5.87	3.50	23.00	9.00	13.00	Z = 2.6132 *p* = 0.0089
	width (II measurement)	9.67	9.30	5.15	3.00	21.00	8.70	10.00	
G2	thickness (I measurement)	3.37	2.90	1.68	1.50	7.50	2.05	4.45	Z = 3.0237 *p* = 0.0025
	thickness (II measurement)	2.59	2.20	1.26	1.00	5.50	1.63	3.20	
	width (I measurement)	7.17	5.10	7.25	2.70	31.00	3.10	7.50	Z = 2.4768 *p* = 0.0133
	width (II measurement)	6.61	4.65	6.54	2.40	28.00	3.10	7.15	
G3	thickness (I measurement)	6.17	4.00	4.39	2.90	15.00	3.35	7.30	Z = 2.2860 *p* = 0.0222
	thickness (II measurement)	4.17	3.00	2.65	2.50	10.00	2.85	4.00	
	width (I measurement)	5.27	3.30	3.83	2.60	12.50	2.75	6.50	Z = 2.2860 *p* = 0.0222
	width (II measurement)	4.33	3.00	3.41	2.00	11.50	2.15	4.75	

## Data Availability

The original contributions presented in this study are included in the article. Further inquiries can be directed to the corresponding author.

## Abbreviations

KT	Kinesiology taping
USG	Ultrasound
VSS	Vancouver Scar Scale
Fig.	Figure
Tab.	Table
